# AAV-Dominant Negative Tumor Necrosis Factor (DN-TNF) Gene Transfer to the Striatum Does Not Rescue Medium Spiny Neurons in the YAC128 Mouse Model of Huntington's Disease

**DOI:** 10.1371/journal.pone.0096544

**Published:** 2014-05-13

**Authors:** Laura Taylor Alto, Xi Chen, Kelly A. Ruhn, Isaac Treviño, Malú G. Tansey

**Affiliations:** 1 Department of Physiology, The University of Texas Southwestern Medical Center, Dallas, Texas, United States of America; 2 Department of Physiology, Emory University School of Medicine, Atlanta, Georgia, United States of America; Univ. Kentucky, United States of America

## Abstract

CNS inflammation is a hallmark of neurodegenerative disease, and recent studies suggest that the inflammatory response may contribute to neuronal demise. In particular, increased tumor necrosis factor (TNF) signaling is implicated in the pathology of both Parkinson's disease (PD) and Alzheimer's disease (AD). We have previously shown that localized gene delivery of dominant negative TNF to the degenerating brain region can limit pathology in animal models of PD and AD. TNF is upregulated in Huntington's disease (HD), like in PD and AD, but it is unknown whether TNF signaling contributes to neuronal degeneration in HD. We used *in vivo* gene delivery to test whether selective reduction of soluble TNF signaling could attenuate medium spiny neuron (MSN) degeneration in the YAC128 transgenic (TG) mouse model of Huntington's disease (HD). AAV vectors encoding cDNA for dominant-negative tumor necrosis factor (DN-TNF) or GFP (control) were injected into the striatum of young adult wild type WT and YAC128 TG mice and achieved 30–50% target coverage. Expression of dominant negative TNF protein was confirmed immunohistologically and biochemically and was maintained as mice aged to one year, but declined significantly over time. However, the extent of striatal DN-TNF gene transfer achieved in our studies was not sufficient to achieve robust effects on neuroinflammation, rescue degenerating MSNs or improve motor function in treated mice. Our findings suggest that alternative drug delivery strategies should be explored to determine whether greater target coverage by DN-TNF protein might afford some level of neuroprotection against HD-like pathology and/or that soluble TNF signaling may not be the primary driver of striatal neuroinflammation and MSN loss in YAC128 TG mice.

## Introduction

Huntington's disease (HD) is an autosomal dominant neurodegenerative disorder caused by CAG repeat expansions in the *huntingtin* gene (htt) that result in an increased number of glutamine residues in the huntingtin protein (polyglutamine expansion). Mutant htt causes degeneration of GABAergic medium spiny neurons (MSN), predominantly in the caudate putamen and cortex, leading to uncontrolled movements, emotional disturbances, dementia and death. A number of cell-autonomous and non-cell-autonomous effects of mutant htt have been documented, suggesting that the mechanisms underlying HD pathology are complex [1].

Chronic neuroinflammation is a characteristic feature of neurodegenerative disorders such as Alzheimer's disease (AD), Parkinson's disease (PD), and amyotrophic lateral sclerosis (ALS) [2], [3] and recent data has established that inflammation is also present in HD [4]. Studies of HD patients have demonstrated that microglia become activated in the brain during disease progression, particularly in regions most affected by neurodegeneration [5]–[8]. In addition, inflammatory gene expression, including Tumor Necrosis Factor (TNF), is increased in brains of mutant htt carriers [9]–[12]. HD patients also have increased immune activation systemically, as evidenced by increased levels of inflammation-related proteins, including TNF, in plasma samples [10], [13]–[15]. Inflammation is also evident in the brain and peripheral blood in mouse models of HD that express mutant htt, including R6/2, YAC128 and HdhQ150/Q150 mice. These animal models have increased microglial density in the brain [16] and elevated expression of inflammation-related genes, including those related to TNF signaling pathways [10], [17].

While pathological effects of inflammation have been established in other models of neurodegenerative disease [2], [3], it still remains to be determined whether inflammatory events driven by TNF contribute to degeneration of MSNs during the progression of HD. We previously demonstrated that blocking soluble TNF signaling using dominant negative TNF (DN-TNF) molecules in animal models of AD and PD attenuates pathology and neurodegeneration [18]–[20]. MSN-like striatally derived cell lines [21] are susceptible to TNF-induced apoptosis *in vitro* [22], [23], suggesting that increased TNF levels in the striatum could be directly toxic to MSNs through activation of pro-apoptotic signaling pathways. In addition, elevated TNF levels could have an indirect effect on neurons by increasing microglial activation, which can result in increased release of cytotoxic substances [24]–[26]. Given that TNF is upregulated in the serum of HD patients and in HD brains post mortem [9], [10], we hypothesized that it plays a critical role in degeneration of MSNs; therefore, we predicted that DN-TNF might protect MSNs by reducing TNF-dependent toxicity on MSNs or by reducing the overall inflammatory state of the striatum. To test this hypothesis, we used adeno-associated virus (AAV) to selectively target soluble TNF via delivery of DN-TNF molecules to the striatum in the YAC128 mouse model of HD.

The YAC128 mouse genome contains a yeast artificial chromosome (YAC) that carries the entire human htt gene, including a 120 CAG repeat mutation [27]. This model has been shown to recapitulate several features of HD pathology, including slowly progressing striatal neuron loss, motor deficits [27]–[35] and increased inflammatory gene expression at 12 months of age [10].

## Methods

### AAV vector production

The human full-length cDNA sequence required for generating DN-TNF protein (TNF variant A145R/I97T) in mammalian cells [36] was kindly provided byXencor, Inc. (Monrovia, CA). It includes the signal peptide sequence required for membrane insertion and the TNF-alpha-converting enzyme (TACE) recognition sequence required for natural cleavage and extracellular secretion. Recombinant AAV plasmid, pTR-UF-12.1 AAV2, was obtained from Dr. Mingji Li (Hope Center Viral Vectors Core, Washington University, MO). The rAAV plasmid contains a multiple cloning site downstream of the cytomegalovirus/chicken beta actin hybrid (CAG) promoter followed by an internal ribosome entry site (IRES) for expression of eGFP. The DN-TNF sequence was cloned into the multiple cloning site of the rAAV plasmid pTR-UF-12.1 AAV2 using HindIII and ClaI. Plasmid pTR-UF-12.1 AAV2 without DN-TNF was used as a control vector (See Figure S1A).

AAV2 serotype 1 viruses were produced using Human Embryonic Kidney (HEK) 293 cells for packaging. Cells were maintained in Dulbecco's modified Eagles medium (DMEM), supplemented with 5% fetal bovine serum (FBS), 100 units/mL penicillin, 100 µg/ml streptomycin in 37°C incubator with 5% CO_2_. The cells were plated at 30–40% confluence in CellSTACKs (Corning) 24 h before transfection (70–80% confluence when transfection). 1.8 mg helper plasmid pXYZ1 (AAV helper plasmid for serotype 1) and 0.6 mg rAAV transfer plasmid were co-transfected into HEK293 cells in a 10-CellSTACK culture chamber (Corning) using the calcium phosphate precipitation procedure [37]. After incubation at 37°C for 3 days, cells were lysed by three freeze/thaw cycles. The cell lysate was treated with 50 U/ml of Benzonase followed by iodixanol gradient centrifugation. The iodixanol gradient fraction was further purified by HiTrap SP column chromatography (GE Healthcare). The eluate was then concentrated with a Centricon Plus-20 100K concentrator (Millipore). Vector titer was determined by Dot blot assay. AAV-DN-TNF-GFP virus titers were 3.3×10^12^–6×10^12^ vg (vector genomes)/mL and AAV-GFP virus titers were 6.4×10^11^–7.3×10^12^ vg/mL.

### Biological activity of DN-TNF protein

Cytoplasmic-to-nuclear translocation of the p65RelA subunit of the NFkB complex was used as a measure of TNF signaling activation. HEK293 cells were plated at the density of 0.02 million cells/well in a 12-well plate. Once cells attached, 2 µL AAV-GFP or AAV-DN-TNF were added to the 1 mL culture medium. After incubation for 3 days, the cells were treated with 10 ng/mL mouse TNF (R&D Systems) (or saline) for 30 minutes, then washed with PBS and fixed with 4% paraformaldehyde. Immunocytochemical staining was performed using anti-p65 primary antibody (Cell Signaling Technology #8242) and Alexa Fluor 594 secondary antibody (Invitrogen A11012).

### Animal care

Generation and breeding of YAC128 HD transgenic mice (FVBN/NJ background strain) has been described previously [27]. Heterozygous YAC128 mice were crossed with wild-type (WT) mice, and the resulting litters were collected, genotyped [38] and housed in pathogen-free climate-controlled facilities at the Animal Resources Center at The University of Texas Southwestern Medical Center at Dallas in Dallas, TX. All animal studies were approved by the Institutional Animal Care and Use Committee at The University of Texas Southwestern Medical Center.

### Vector injection surgery

2 month old animals (**Table 1**) were anesthetized with 50 mg/kg ketamine, 5 mg/kg xylazine and 1 mg/kg acepromazine before being placed in a stereotaxic frame. AAV-DN-TNF-GFP or AAV-GFP (negative control) vectors were injected bilaterally into the striatum through burr holes in the skull (stereotaxic injection coordinates relative to Bregma: anterior/posterior +1.3, mediolateral +2.2, dorsoventral −2.8). Vector solution (3 µL/side) was injected at 0.5 µL/min. using a Hamilton syringe. The syringe was left in place for 3 min. after all liquid had been dispensed and then slowly withdrawn over 3 min. Animals received buprenex (buprenorphine hydrochloride, 6 µg/injection) postoperatively for 3 days.

**Table 1:** Number of naive animals used for each type of experimental analysis.

Group	qPCR (3 mo.)	qPCR (9 mo.)	Motor function, qPCR (12 mo.)	Serum ELISA (12 mo.)	Microglia quantification (9 mo.)	Motor function, Microglia quantification (12 mo.)	Total
WT	3	5	4	6	5	5	28
TG	3	3	4	4	5	5	24

Wild type or YAC128 transgenic (TG) male and female mice that received no virus injections were compared by quantitative PCR at 3, 9 and 12 months. Additional 12-month animals were used for serum multiplex ELISA. The same animals used for quantitative PCR (qPCR) and microglia quantification at 12 months were also used for assessment of motor function. WT, wild type; TG, transgenic.

### Rotarod and beamwalk functional tests

Mice underwent behavioral testing on balance beam and rotarod apparatus (Economex 0207-005 M; Columbus Instruments) at 3, 5, 7, 9, 11 and 12 months of age. At each time point, mice were trained for 3 days on each task followed by testing on the fourth day. Training and testing were done at the same time each day. Beamwalk training consisted of 4 traverses across a 17-mm round beam into a home cage. On the test day, mice were required to traverse 3 different beams (17 mm round, 11 mm round and 5 mm square beams) into a home cage with 3 trials on each beam. The 3 trials were done consecutively with 30 min. of rest between different beam sizes. Average time to traverse the beam was calculated and used to compare groups. Rotarod training consisted of 3 runs in which mice were placed on an accelerating rotarod (0.2 rpm/s) until they fell from the rod. The test day consisted of 3 trials running on the accelerating rotarod with an inter-trial interval of 30 min. Average time spent running on the rod was measured and used to compare between groups.

### DN-TNF ELISA

A human TNF-specific ELISA can be used to detect expression of DN-TNF without cross-reactivity with endogenous murine TNF. Mice were given a lethal dose of Euthasol (Burns Vet supply), decapitated and the left and right striata were dissected, weighed and immediately flash frozen in liquid nitrogen. Right samples were sonicated in 20 µL/mg of cold lysis buffer containing a protease inhibitor cocktail (Roche Diagnostics, Mannheim, Germany), centrifuged at 4°C and used for huTNF-specific ELISA according to manufacturer's instructions (HuTNFα Cytoset, Biosource International, Camarillo, CA).

### Quantitative PCR

Left striatum samples (n = 3–5 animals per group) were collected and flash frozen as described above and homogenized in ice-cold RLT buffer (Qiagen, Valencia, CA) containing 2-mercaptoethanol using a rotor-stator homogenizer followed by centrifugation in QIAshredder spin columns (Qiagen). RNA was isolated using the Qiagen RNeasy kit (Qiagen, Valencia, CA) according to manufacturer's instructions, treated with DNase I (Invitrogen, Carlsbad, CA), and reverse transcribed using Superscript II RNase H-reverse transcriptase (Invitrogen). RNA concentration was determined by absorbance at 260 nm using a Nanodrop (Thermo Scientific, Wilmington, DE). Oligonucleotide primers for gene amplification of murine TNF, interleukin-6 (IL-6), chemokine (C-C motif) ligand 2 (CCL2, MCP1), chemokine (C-C motif) ligand 5 (CCL5, RANTES), protein tyrosine phosphatase receptor type C (PTPRC, CD45), glial fibrillary acidic protein (GFAP), CD68, chemokine (C-C motif) ligand 3 (CCL3, MIP1a), inducible nitric oxide synthase (iNOS), chemokine (C-X-C motif) ligand 2 (MIP2), interferon gamma (IFNγ), interleukin-4 (IL-4), and interleukin-10 (IL-10) were obtained from Integrated DNA Technologies and validated by analysis of template titration and dissociation curves (primer sequences available on request). Quantitative real-time PCR was performed using an ABI Prism 7900HT Fast Detection System (Applied Biosystems). Each 10 µL reaction was performed in 384-well format with 25 ng of cDNA, 5 µL of *Power* SYBR green PCR Master Mix (Applied Biosystems) and a concentration of 0.15 µM of each PCR primer. All reactions were performed in triplicate and average values were used. mRNA expression levels were normalized to those of the mouse housekeeping gene glyceraldehyde-3-phosphate dehydrogenase (GAPDH), which was found to be unchanged by genotype or treatment, and evaluated by the comparative CT method (User Bulletin No. 2, Perkin Elmer Life Sciences). Values were normalized to 3 month old WT animals and compared by 2-way ANOVA (genotype, age) followed by Bonferroni posthoc tests.

### Multiplexed ELISA

Levels of murine interferon-gamma (IFNγ), interleukin-1 beta (IL-1β), interleukin-6 (IL-6), interleukin-10 (IL-10), interleukin-12p70 (IL-12p70), chemokine (C-X-C motif) ligand 1 (CXCL1, KC) and tumor necrosis factor (TNF) in serum were analyzed using a multiplex assay according to manufacturer's instructions (Meso Scale Discovery, Gaithersburg, MD). For serum collection, animals were deeply anesthetized with ketamine/xylazine cocktail (described above), then decapitated. Blood was collected in vacutainers (Sarstedt Inc., Newton, NC) and spun at 13,000 rpm for 10 min. The serum fraction was removed and frozen at −80°C. Serum samples from individual animals were used in the multiplexed immunoassay.

### Stereological quantification of NeuN positive neurons

Animals used for quantification of MSNs were the same animals used for behavioral testing. After transcardial perfusion with 12.5 mL ice cold PBS supplemented with 0.1% glucose and 0.1% heparin followed by 50 mL 4% paraformaldehyde (PFA) in PBS, dissected brains were post-fixed in 4% PFA for 24 h at 4°C, equilibrated in 20–30% sucrose in PBS for 24–48 h at 4°C and stored in PBS plus 0.01% sodium azide at 4°C. The brains were then sliced into 30 µm-thick coronal sections using a SM2010R sliding microtome (Leica, Bannockburn, IL). Coronal sections spaced 360 µm apart throughout the striatum (+1.70 mm to −2.30 mm relative to Bregma) were collected. For quantification of neuronal loss, sections were stained with anti-NeuN monoclonal antibody (1∶1000 dilution; Millipore, Billerica, MA) using a free-floating protocol. Biotinylated anti-mouse IgG reagent was used as the secondary antibody (1∶250 dilution; M.O.M. kit, Vector Laboratories, Burlingame, CA). Signal was amplified with a Vectastain *Elite* ABC kit (Vector Laboratories) and detected with diaminobenzidine (DAB) (Vector Laboratories). Labeled sections were mounted on slides and coverslipped using Glycergel Mounting Medium (Dako, Carpinteria, CA). Stereo Investigator analysis software (Micro Bright Field, Williston, VT) was used for carrying out unbiased stereological counts of NeuN-positive neuronal nuclei in the striatum, employing the optical fractionator method [39]. All analyses were performed blindly with respect to the nature of slices (genotype and AAV injection). The grid size was set to 450×450 µm, and the counting frame was set to 50×50 µm. The average slice thickness after histological processing was determined to be 22–23 µm.

### Quantification of activated microglia in the striatum

Tissue was prepared for labeling as described for stereological quantification of NeuN positive neurons above. Coronal sections spaced 360 µm apart throughout the striatum (+1.70 mm to −2.30 mm relative to Bregma) were collected and stained with goat polyclonal anti-Iba1 antibody (1∶600 dilution; Abcam, Cambridge, MA) using a free-floating protocol. Alexa Fluoro 594 goat anti-rabbit IgG (1∶1000 dilution; Invitrogen, Carlsbad, CA) was used as the secondary antibody. Cover slips were applied to mounted brain sections using Fluoro-Gel anti-fade (Electron Microscopy Sciences). Serial fluorescent images were captured and the number of striatal microglia per field (field size 832×666 µm, width x height, 1 field per section) was quantified by using Nikon NIS-Element software.

### Stereological quantification of AAV-GFP vector transduction efficiency

Coronal sections spaced 360 µm apart throughout the striatum (+1.70 mm to −2.30 mm relative to Bregma) were collected from 5 randomly chosen wild type animals that received AAV-GFP vector injections and mounted onto slides. The volume of the striatum that contained GFP-labeled cells was estimated using the Cavalieri Estimator Probe of the Stereo Investigator analysis software. The grid size was set to 100 µm.

### Statistics

T-tests, one-way, and two-way ANOVA's were performed using GraphPad Prism software version 6.0 for Mac, GraphPad Software, La Jolla CA. Two-way repeated measures ANOVA's were performed using SPSS (IBM Corp. Released 2011. IBM SPSS Statistics for Mac, Version 20.0. Armonk, NY: IBM Corp. t-tests were used to assess potential differences in serum cytokines between wild type and transgenic mice. Two way ANOVAs were used to examine potential differences between genotype and age and genotype and AAV-injection. A two way repeated measures ANOVA was used to determine the effect of genotype and AAV-injection on the development of behavioral dysfunction over time. A repeated measures ANOVA was performed to examine the effect of genotype in the development of behavior over time. When sphericity was not met (p>0.05), Greenhouse-Geissen test was used (Epsilon <0.75) to calculate the appropriate adjustment to degrees of freedom of the F-statistic. Statistics are reported in the figure legends. Asterisks indicate the results of the post hoc tests following a significant interaction.

### Ethics Statement

All animal studies were approved by the Institutional Animal Care and Use Committee at The University of Texas Southwestern Medical Center.

## Results

### YAC128 transgenic mice display neuroinflammation at 12 months of age

**Figure 1:**
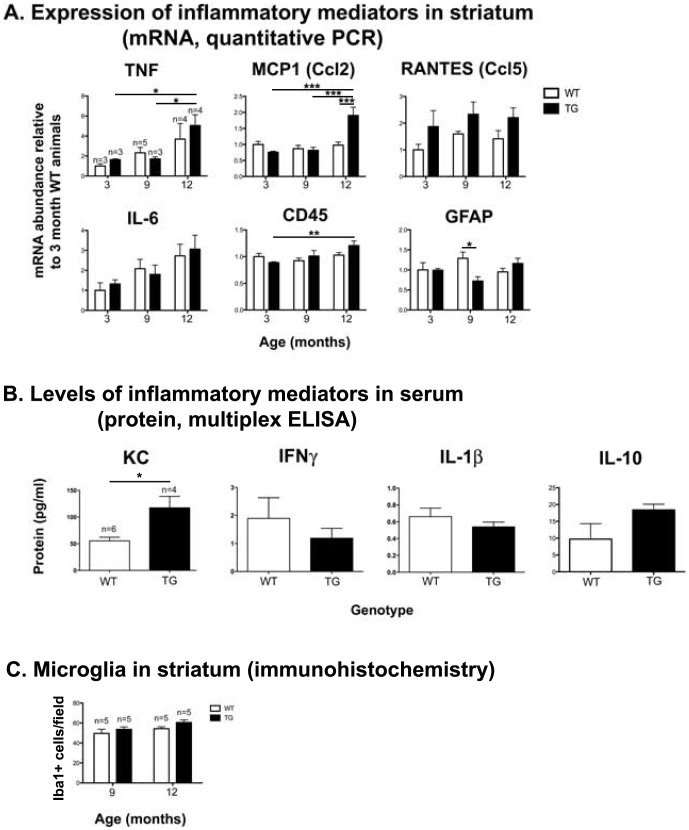
YAC128 TG mice display a moderately increased neuroinflammatory profile relative to WT mice. (A) Quantitative PCR analysis using mRNA isolated from dissected striatum in unmanipulated YAC128 transgenic (TG) and WT male and female mice age 3, 9 and 12 months, relative to 3-month old WT animals. There is a significant interaction between age and genotype for MCP1 and GFAP (p = 0.0019, and p = 0.03, respectively), a significant effect of age for TNF, IL-6, and CD45 (p = 0.01, p = 0.02 and p = 0.03, respectively) and a significant effect of genotype for RANTES (p = 0.01). 2-way ANOVA, Bonferroni's post test, *p<0.05, **p<0.01, ***p<0.001. (B) Multiplex ELISA for inflammatory mediators in serum samples taken from 12-month un-operated mice (t-test, *p<0.05). (C) Quantification of activated microglia in the striatum in un-operated mice age 9 and 12 months. 2-way ANOVA (genotype, age) revealed no differences between groups.

To provide rationale for an anti-inflammatory intervention to rescue MSNs, we first assessed the inflammatory profile of YAC128 transgenic (TG) compared to wild type (WT) mice using three independent approaches. First, we measured mRNA levels of inflammation-related genes in striata of naive mice as they aged over 12 months (**Fig. 1A**, **Table 2**). TNF gene expression increased with age in both WT and TG mice. MCP1 on the other hand, was significantly increased at 12 months in TG mice only. RANTES expression was increased in both young and aged TG striata compared with WT striata (significant effect of genotype), while IL-6 increased over time in striata from mice of both genotypes (significant effect of age). We observed small (less than 0.5-fold) changes in expression of the glial activation markers CD45 and GFAP. Expression of the pan-microglial marker CD68 and inflammatory mediators MIP1a and iNOS did not change significantly between genotypes or with age (data not shown). MIP2, IFNγ, IL-4 and IL-10 mRNA transcripts were not detectable in the striatum in mice of either genotype at any time point (data not shown).

**Table 2:** Number of AAV-GFP or AAV-DN-TNF animals used for each type of experimental analysis.

Group	DN-TNF ELISA	DN-TNF ELISA	DN-TNF ELISA, qPCR,	Motor function,	Total
	(3 mo.)	(9 mo.)	Serum ELISA	MSN	
			(12 mo.)	count (12 mo.)	
WT+AAV-GFP	3		5	9	17
WT+AAV-DN-TNF	3		4	8	15
TG+AAV-GFP	3	3	5	12	23
TG+AAV-DN-TNF	3	3	4	9	19

Animals from 4 treatment groups (column 1) were used for DN-TNF ELISA at 3, 9 and 12 months of age. 12-month animals were also used for quantitative PCR (qPCR) and serum multiplex ELISA (Serum ELISA). An additional group of animals was used for assessment of motor function and quantification of MSNs (MSN count) in the striatum. WT, wild type; TG, transgenic.

To obtain a second measure of inflammation in YAC128 TG mice, we quantified the levels of inflammation-related proteins in serum from 12-month-old YAC128 TG and WT animals using multiplexed ELISA (**Fig. 1B**). In serum, KC levels were significantly increased in transgenic mice while IL-10, IFNγ and IL-1β levels were not significantly different between genotypes. IL-6, TNF and IL-12p70 levels were not detectable.

As a third measure of the inflammatory profile of YAC128 TG mice, we quantified activated microglia in the striatum using Iba1 immunohistochemistry (**Fig. 1C**). We found no difference between genotypes in the number of activated microglia at either 9 or 12 months. Taken together, these data suggested that aged YAC128 TG mice had a moderately increased inflammatory profile compared to WT mice at 12 months of age.

### *In vitro* infection with AAV-DN-TNF blocks TNF-dependent NFκB signaling

We generated AAV vectors that expressed either GFP alone (negative control) or dominant negative TNF (DN-TNF [36], [40]) and GFP, with GFP expressed from an internal ribosome entry site (**Fig. S1A**). To confirm that the AAV-derived DN-TNF was bioactive and capable of blocking soluble TNF signal transduction, we infected HEK293 cells and measured TNF-induced cytoplasmic-to-nuclear translocation of the p65RelA subunit of NFkB *in vitro* using immunocytochemistry. GFP fluorescence confirmed high infectivity (data not shown) and cells infected with AAV-DN-TNF displayed minimal nuclear enrichment of p65, whereas AAV-GFP infected cells displayed strong nuclear enrichment of p65 (**Fig. S1B**).

### Bilateral *in vivo* injections of AAV-DN-TNF result in limited coverage of mouse striatum and transgene silencing over time

**Figure 2:**
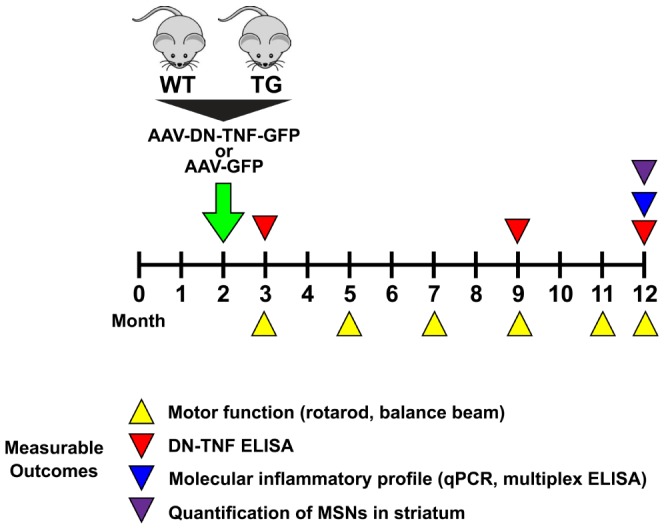
Experimental Design. YAC128 TG and WT male and female mice received injections of AAV-DN-TNF-GFP (GFP is expressed from an IRES) or AAV-GFP (control) in the striatum at 2 months of age (green arrow). A cohort of animals underwent motor function testing on balance beam and rotarod (yellow arrowheads) at 3, 5, 7, 9, 11 and 12 months. Then, medium spiny neurons in the striatum were quantified by unbiased stereology (black arrowhead). At 3, 9 and 12 months of age, additional cohorts of animals were used to measure DN-TNF protein in the striatum by ELISA (red arrowheads) and for analysis of inflammation-related gene expression by qPCR and protein by multiplexed ELISA (blue arrowhead).

**Figure 3:**
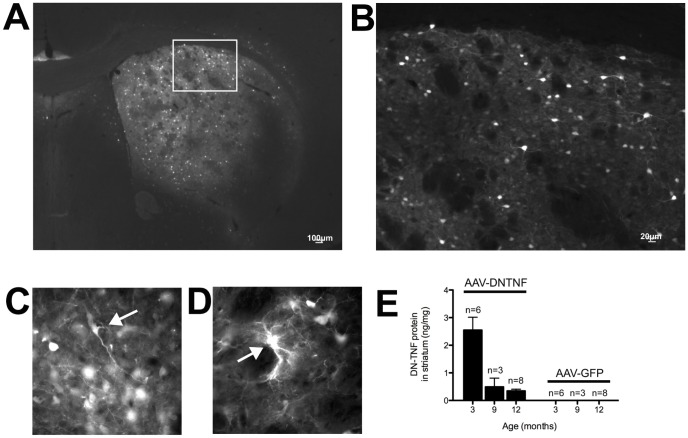
AAV-DN-TNF-GFP and AAV-GFP vector injections resulted in sustained transgene expression in the striatum. (A) GFP expression in a 12-month animal that received AAV-GFP vector injection. (B) Higher magnification view of boxed area. AAV vectors transduced (C) neurons and (D) glia (arrows). (E) DN-TNF ELISA using dissected striata from 3, 9 and 12-month animals indicates long-term expression of the DN-TNF transgene only in animals that received DN-TNF vectors.

At 2 months of age WT and TG mice received injections of AAV viral stocks into the striatum (**Fig. 2** **,** **Table 2**). Analysis of GFP expression in 12-month animals injected with AAV vectors revealed the presence of numerous GFP-expressing cells scattered throughout the dorsomedial aspect of the striatum in aged animals (**Fig. 3A,B**). Based on the morphology of GFP-expressing cells we concluded that AAV vectors infected primarily neurons but also cells likely to be glia, (**Fig. 3C,D**). It should be noted, however, that stereological quantification of GFP-expressing cells revealed that *in vivo* transduction with AAV-GFP achieved coverage of ∼30–50% of striatal volume in aged animals, indicating that not all striatal neurons were maintained in an environment of increased DN-TNF expression throughout the study. To directly quantify DN-TNF levels, we used a human TNF-specific ELISA that specifically recognizes the DN-TNF human protein sequence but not endogenous mouse TNF (**Fig. 3E**). DN-TNF levels in striatal tissue decreased over time, but were well above background levels at all time points tested. There was no significant difference between genotypes in DN-TNF levels achieved after transduction with AAV-DN-TNF (data not shown).

### Bilateral *in vivo* injections of AAV-DN-TNF fail to rescue MSN degeneration in the striatum of YAC128 TG mice

**Figure 4:**
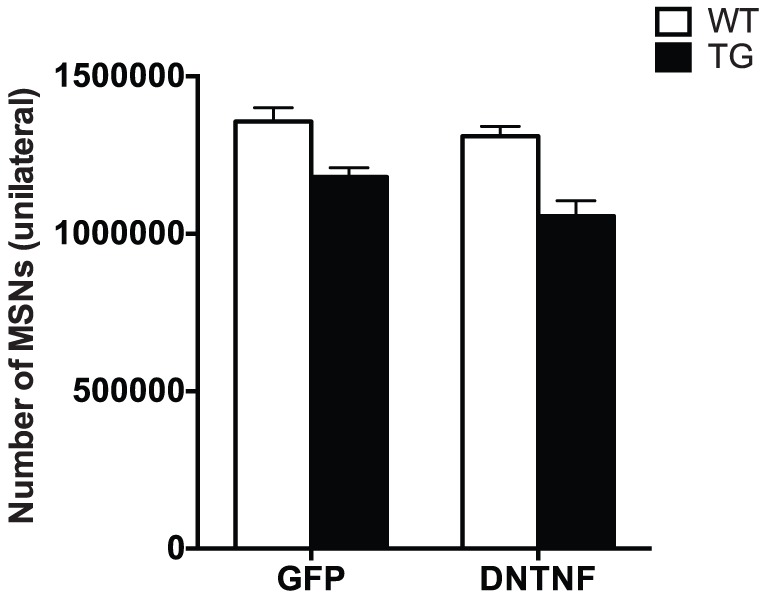
AAV-DN-TNF gene transfer in the striatum did not reduce MSN degeneration in YAC128 TG mice. NeuN-positive neuronal nuclei in striata of 12-month animals were quantified using unbiased stereology and compared across treatment groups. YAC128 TG mice had significantly less neurons (main effect of genotype F(1,34) = 4.7, p<0.05) that were not affected by AAV-DNTNF injection (no significant interaction: F(1,34) = 0.99, p>0.05).

After 12 months, TG and WT animals that had received injections of AAV-DN-TNF or AAV-GFP control vectors at 2 months of age were euthanized and MSN number in the striatum was quantified by unbiased stereology (**Fig. 4** **,** **Table 2**). Consistent with previous findings, YAC128 TG mice had fewer striatal MSNs compared to WT animals. However, in contrast to our previous findings in PD and AD rodent models in which DN-TNF peptide was directly infused [18], [19] or expressed via lentivirus [18], [20], [41], single bilateral injections of AAV-DN-TNF delivery did not rescue degenerating MSNs in YAC128 TG mice compared to AAV-GFP.

### Bilateral *in vivo* injections of AAV-DN-TNF fail to reduce age-dependent locomotor deficits in YAC128 mice

**Figure 5:**
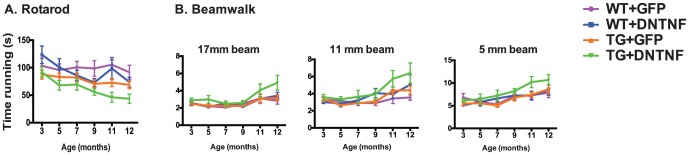
AAV-DN-TNF gene transfer did not rescue motor function deficits in YAC128 TG mice. AAV-injected animals were evaluated in tests of motor function including (A) time running on the accelerating rotarod and (B) time to cross a 17 mm, 11 mm and 5 mm diameter beam. A two-way repeated measures ANOVA indicated no statistically significant interaction between genotype and virus in motor performance across time. Rotorod: F(5,170) = 0.798, p = 0.378; Beamwalk 17 mm: F(5,170) = 1.03, p = 0.621, Beamwalk 11 mm: F(5,170) = 0.794, p = 0.850; Beamwalk 5 mm F(5,170) = 0.335, p = 0.818.

Previous studies have demonstrated that YAC128 TG mice perform worse in tests of motor skills [27]–[33]. Our own preliminary studies in un-operated YAC128 and WT mice confirmed this deficit, demonstrating that at 12 months of age YAC128 transgenic mice performed significantly worse in balance beam and rotarod tests than WT mice (**Fig. S2A,B**). Locomotor performance was not improved in YAC128 TG mice following AAV-DN-TNF injection (**Fig. 5A,B** **,** **Table 2**). In addition, analysis of gait and stride parameters were conducted on the DigiGait (Mouse Specifics) but no significant effects of AAV-DN-TNF were detected (data not shown). The lack of locomotor improvement by AAV-DN-TNF was consistent with anatomical data showing that AAV-DN-TNF delivery did not rescue MSNs in YAC128 TG mice (**Fig. 4**).

### Bilateral *in vivo* injections of AAV-DN-TNF fail to exert robust anti-inflammatory effects in YAC128 TG mice

**Figure 6:**
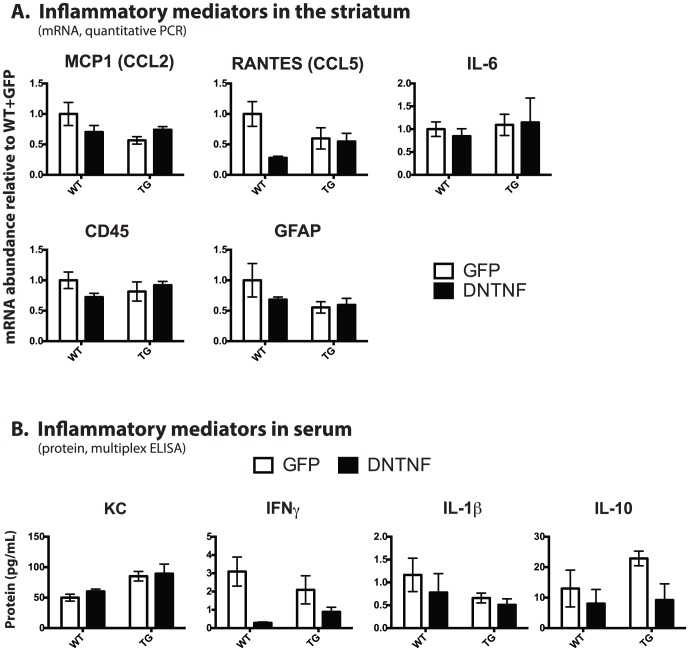
AAV-DN-TNF did not significantly reduce markers of inflammation in YAC128 TG mice. 12-month TG or WT male and female mice that received AAV-DN-TNF or AAV-GFP viral vector injections at 2 months were compared using (A) quantitative PCR to measure expression of inflammatory markers in dissected striata. Mice injected with AAV-DNTNF had reduced CCL5 compared to those injected with AAV-GFP, regardless of genotype (main effect of AAV-injection: F(1,14) = 5.6, p<0.05). (B) Levels of inflammation-related proteins in serum in the same animals were compared by multiplexed ELISA. Transgenic mice had increased KC, regardless of AAV-injection (main effect: F(1,14), 12.7, p<0.05) whereas DNTNF reduced IFNγ protein levels in both WT and transgenic mice (main effect of AAV-injection: F(1,14) = 10, p<0.05). 2-way ANOVA.

To determine whether long-term AAV-DN-TNF delivery was able to reduce inflammatory marker expression in aged YAC128 TG mice, we again measured inflammation-related gene expression in the striatum (**Fig. 6A** **,** **Table 2**) and protein levels in serum (**Fig. 6B**) in aged mice that received AAV vectors. In general, expression of inflammation-related genes in the striatum was not changed significantly in animals treated with AAV-DN-TNF compared with AAV-GFP (**Fig. 6A**). One exception was RANTES, for which mRNA levels were moderately but significantly reduced by AAV-DN-TNF injection. Serum levels of IFNγ, IL-1β and IL-10 displayed slight reductions in animals that received striatal AAV-DN-TNF injections compared to animals that received striatal AAV-GFP injections, but in general the reduction was not statistically significant (**Fig. 6B**). Serum IL-6 and IL-12p70 levels were below the level of detection. Lastly, measurements of endogenous TNF protein was excluded from the analysis because of possible contamination by DN-TNF heterotrimers with native TNF.

## Discussion

HD is a neurodegenerative disorder that is characterized neuropathologically by loss of medium spiny neurons in the striatum. Inflammation in the central nervous system is thought to contribute to neuronal death in several neurodegenerative diseases and targeting these processes as a way to decrease or slow the death of neurons has emerged as a possible therapeutic option [3], [4]. It has now become clear that inflammation is also at play in HD, but the contribution of inflammatory processes to pathology in HD is just beginning to be explored [4].

Here, we report that YAC128 mice exhibited moderate age-related increases in inflammatory markers, confirming previous reports [10], [42]. In this study, we tested the efficacy of AAV-DN-TNF gene transfer targeted to the striatum in reducing neuroinflammation and/or attenuating neurodegeneration of MSNs in the YAC128 HD model. However, despite confirmation that bioactive DN-TNF was encoded and produced by cells infected with AAV-DN-TNF *in vitro*, bilateral single injections of AAV-DN-TNF into the striatum had limited effects on all outcome measures. Given that immunohistological analyses indicated 30–50% of the striatum was transduced by the single bilateral injections, one explanation for the limited efficacy was poor target coverage of the striatum, a rather large anatomical structure. Even if this extent of target coverage had been sufficient to achieve a positive outcome, a second contributing factor may have been the decline in DN-TNF expression over time which is likely to be related to transgene silencing, a common occurrence in viral-mediated gene transfer [43]. Given these technical challenges, we can only conclude that transduction of up to 50% of the striatum with AAV-DN-TNF is not sufficient to inhibit age-dependent neuroinflammation, locomotor deficits, and loss of MSN number in YAC128 mice. It is entirely possible that if target coverage had been greater and DN-TNF production had remained stable at the levels measured in 3-month old animals throughout the study, significant attenuation of neuroinflammation, locomotor deficits, and MSN degeneration might have been achieved.

Despite reduced transgene expression over time, an argument could be made that even with only 30–50% coverage of the striatum remaining at 12 months, some attenuation of HD-like pathology should have been detectable in YAC128 mice if soluble TNF were a critical driver of neuroinflammation and MSN death, especially given that we measured increased DN-TNF levels by ELISA in 12-month animals. In fact, DN-TNF levels were 10-fold higher (per mg tissue) than those measured in the substantia nigra after delivery of DN-TNF via lentivirus, a treatment that was effective in reducing dopaminergic neuron death in a striatal 6-hydroxydopamine model of PD [20].

Few studies have addressed whether inflammation contributes to neuronal death in HD, but several reports in rodent models of HD-like pathology have provided evidence that it may indeed play a role. For example, one recent report showed that microglial CB(2) cannabinoid receptor signaling reduced microglial activation and neuroinflammation and prevented neuron loss in a mouse model of intrastriatal quinolinic acid-induced excitotoxicity [44]. These findings suggest that microglial activation, a hallmark of CNS inflammation, contributes to neuron death. Interestingly, treatment with the tetracycline antibiotic, minocycline, resulted in delayed disease progression in the R6/2 mouse model [23], [45], [46]. Minocycline is thought to have anti-inflammatory effects, such as reduced microglial activation in response to injury or neurotoxin exposure [47], [48]. Thus, minocycline treatment in the R6/2 mouse was reported to delay disease progression, at least in part, by attenuation of the inflammatory response. Taken together, previous published studies suggested that inhibition of soluble TNF should have attenuated neuroinflammatory responses and rescued MSN neurons in the YAC128 model. As mentioned above, alternative delivery strategies to ensure greater anatomical target (striatum) coverage merit investigation to successfully validate soluble TNF as a target in HD models.

One emerging hypothesis is that mutant htt expression causes immune cell dysfunction that contributes to CNS pathology in HD. Kwan et al. [42] showed that transplantation of WT bone marrow cells into mouse models of mutant htt expression (including YAC128 mice), reduced inflammatory mediators in the periphery and conferred some benefit in disease progression. The authors suggested that mutant htt causes impaired cytokine and chemokine signaling, which has been shown to cause chronic increases in these molecules that is similar to what is observed in human patients and animal models of HD [49]–[52]. WT immune cells may help re-establish normal cytokine and chemokine signaling, reducing inflammation and attenuating disease progression. Notably, this study reported that complete normalization of cytokine and chemokine abnormalities in the peripheral blood of HD mice resulted in only a modest reduction in pathology and behavioral deficits. Another recent report demonstrated that long-term peripheral LPS administration, which causes increased CNS inflammation and neuron loss in several mouse models of neurodegeneration [53], did not appear to affect neuron loss in YAC128 mice, despite clearly increased microglial activation [54]. Together, these studies raise the possibility that cytokine/chemokine signaling may play a limited role in the pathology caused by mutant htt in the YAC128 mouse model of HD. Our results demonstrating that selective blocking of soluble TNF in up to 50% of the striatum did not reduce MSN degeneration are consistent with this idea.

We hypothesized that TNF could be directly toxic to CNS neurons via the TNF death receptor signaling pathway–as observed in PD models–or could contribute to an enhanced inflammatory environment in the striatum that would cause activation of MG and release of other cytotoxic molecules. In support of this idea, MSN-like striatally-derived neuronal cell lines are susceptible to TNF-induced apoptosis *in vitro*, suggesting that the signaling machinery for TNF death receptor function is in place [22], [23]. However, it is entirely possible that *in vivo* MSN in the striatum display a different arsenal of inflammatory responses (or are less susceptible to soluble TNF) relative to other regions where we have successfully neutralized soluble TNF, such as the substantia nigra pars compacta (SNpc) and achieved neuroprotection. In support of this idea, chronic infusion of DN-TNF protein into SNpc, but not into striatum, attenuated neuroinflammation and loss of DA neurons in SNpc induced by a striatal 6-hydroxydopamine lesion [19].

What other factors in addition to, or independent of, poor target coverage and transgene silencing might account for the inability of AAV-DN-TNF to rescue MSN in striatum in the YAC128 model of HD? First, TNF levels may not be critical for neuronal death. Post-mortem studies [9], [10] suggest that TNF levels are elevated in HD patient striatum, but not in all HD mouse models [10]. Similarly, we observed no difference between YAC128 TG and WT 12-month animals in striatal TNF levels. 12-month old YAC128 mice represent early stage disease [27], and based on post-mortem studies of human brain, TNF levels correlate with disease severity [9], [10]. Thus, TNF levels may continue to rise with disease progression. If so, TNF pro-apoptotic signaling may contribute to MSN death in this model, but only at late time points when TNF levels may be more substantially increased. In several of the PD and AD preclinical animal models in which targeted delivery of lenti-DN-TNF proved efficacious [18], [19], exogenous neurotoxic or inflammatory stimuli were administered as part of the paradigm to accelerate neuronal degeneration. Specifically, McCoy et al. used an intrastriatal 6-OHDA lesion model and an intranigral LPS model and McAlpine et al. used LPS as a “second hit” in the 3×Tg mouse to accelerate intraneuronal amyloid deposition. In contrast, the inflammation we observed in the YAC128 mouse was a direct consequence of mutant htt expression; there was no additional inflammatory stimulus given. Thus, inflammation in the YAC128 mouse model of HD is likely to develop more slowly and be less severe. Under these circumstances, TNF signaling might not contribute substantially to neuronal death. If soluble TNF were a critical driver of neuroinflammation and MSN death, one might expect at least some attenuation of pathology, despite delivery to a limited portion of the striatum. Second, it has been proposed that HD is associated with a more specific set of inflammatory changes compared to the more generalized chronic neuroinflammation observed in PD or AD [4]. The early and sustained up-regulation of cytokines and chemokines, including TNF, in serum of HD patients [10] seems to be a unique feature of HD inflammation. Since the significance of these changes is not understood, it is possible that TNF signaling plays a role in pathogenesis, but not at the level of MSNs in the striatum. For example, elevated levels of TNF circulating in blood might contribute to over-activation of immune cells that might not be attenuated by a highly localized block of TNF signaling in the striatum. If so, blocking TNF in the systemic circulation may represent an alternative and more efficacious approach to achieve CNS benefits. In support of this idea, subcutaneous administration of a PEGylated form of the DN-TNF protein (XPro1595) in an experimental autoimmune encephalomyelitis (EAE) model of multiple sclerosis attenuated CNS lesions, preserved axons, and promoted remyelination [55].

In summary, we conclude that the YAC128 mouse model displays age-dependent neuroinflammation in the striatum and is therefore an appropriate model in which to explore the role of inflammation in MSN degeneration and HD-like phenotypes. However, our ability to test the hypothesis that soluble TNF plays a critical role in mediating these effects was limited by the inherent drawbacks associated with viral-mediated gene transfer. Specifically, our single bilateral injections of AAV-DN-TNF yielded at best 50% coverage of the target structure (i.e. the striatum) and expression of DN-TNF declined over time. Together, this level of neutralization of soluble TNF could have been insufficient to attenuate age-dependent neuroinflammation, MSN degeneration, and locomotor deficits in the YAC128 mice. On the other hand, these results could indicate that TNF signaling in the brain has different consequences in the context of mutant htt expression than in other neurodegenerative diseases.

Given the technical limitations of our study, we believe these data should not be interpreted to mean that TNF and neuroinflammation in general do not play a role in HD pathophysiology. Instead, our results may highlight the importance of deciphering which inflammatory signals contribute to neuron death and precisely where these signals are important. Several studies have documented increased inflammation in HD by comparing levels of cytokines and chemokines in brain. Quantitative PCR using postmortem HD patient brains revealed a significant increase in IL-10, IL-6, IL-8, MMP9 and CCL2 and a trend toward an increase in TNF in the striatum of HD patients compared to control patients [9]. Highly significant increases (over 1000-fold) in IL-8 and IL-6 transcripts and significant increases in TNF (2-fold) in HD patient post-mortem striatal tissue have been reported in independent studies [10]. In our study, 12-month YAC128 mouse brains had clearly increased striatal levels of MCP1 (CCL2) compared to control mouse brains, similar to human HD patient brain samples [9], [10]. Interestingly, Godavarthi et al. [56] showed that neuro2a cells overexpressing mutant htt expressed increased levels of MCP1 and Kwan et al. reported that monocytes isolated from HD patients were impaired in their ability to migrate toward MCP-1 [57]. We also observed an overall up-regulation of RANTES (CCL5) in YAC128 TG mouse striatum consistent with previous studies showing that RANTES levels may be affected by mutant htt [58]. The specific upregulation of these inflammatory signals may warrant further investigation.

## Conclusions

YAC128 mice display moderate age-related increases in inflammatory markers and thus represent a good model in which to test the role of neuroinflammation in progression of HD-like pathology. While high transduction efficiency of AAV-DN-TNF *in vitro* resulted in inhibition of TNF-dependent NFκB signal transduction, limited AAV-DN-TNF *in vivo* spread after single bilateral striatal injections and decline in DN-TNF protein levels over time may have contributed to the lack of effects on age-related neuroinflammation, MSN loss, and locomotor deficits in YAC128 mice. Given these unfortunate but not uncommon technical limitations of gene transfer, alternative delivery approaches for DN-TNF should be explored to ensure greater coverage of large anatomical structures like the striatum to unequivocally establish whether neutralization of soluble TNF affords therapeutic benefit in this mouse model of HD.

## Supporting Information

Figure S1 **AAV vectors for the expression of dominant negative TNF.** (A) AAV2 construct for expression of DN-TNF and enhanced GFP (eGFP, top), with eGFP expressed from an internal ribosome entry site. AAV2 control construct (bottom) for expression of eGFP alone (ITR, inverted terminal repeat; CAG, chicken β-actin promoter; DN-TNF, dominant negative TNF coding sequence; IRES, internal ribosome entry site; eGFP, enhanced GFP; polyA, bovine growth hormone [bGH] polyA sequence). (B) DN-TNF protein produced by AAV2/1 serotype virus is biologically active. In unstimulated HEK293 cells, p65RelA displays a cytoplasmic distribution regardless of whether they are transduced with AAV-GFP or AAV-DN-TNF (left panels); in cells treated with TNF (right panels), AAV-GFP-transduced cells display translocation of the p65RelA subunit of NFkB to the nucleus (top right), demonstrating that TNF receptors have been activated by TNF binding. In cells transduced with AAV-DN-TNF (bottom right), p65 does not enter the nucleus because soluble TNF has been sequestered away from TNF receptors by dominant negative TNF. Scale bar, 100 µm.
(EPS)

Figure S2 **Aged YAC128 transgenic animals exhibit locomotor deficits in several functional tests.** YAC128 TG and WT male and female mice were compared in tests of motor function including (A) time running on the accelerating rotarod and (B) time to cross a 17 mm, 11 mm and 5 mm diameter at 7, 9 and 12 months. Statistical data for rotarod (A): interaction p = 0.003, genotype p = 0.032, time p = 0.358. Statistical data for beamwalk (B): interaction p = 0.304, p = 0.072, p = 0.007 for 17, 11 and 5 mm beams, respectively; time p = 0.049, p = 0.002, p<0.0001 for 17, 11 and 5 mm beams, respectively; genotype p = 0.048, p = 0.063, p = 0.005 for 17, 11, 5 mm beams, respectively (repeated measures ANOVA, Bonferroni post hoc test). *p<0.05, **p<0.01, ***p<0.001.
(EPS)
